# Hip Osteoarthritis and the Risk of Lacunar Stroke: A Two-Sample Mendelian Randomization Study

**DOI:** 10.3390/genes13091584

**Published:** 2022-09-03

**Authors:** Yi Shen, Fuju Li, Lina Cao, Yunyun Wang, Jing Xiao, Xiaoyi Zhou, Tian Tian

**Affiliations:** 1 Department of Epidemiology & Health Statistics, School of Public Health, Nantong University, Nantong 226019, China; 2 Center for Disease Control and Prevention of Nantong, Nantong 226007, China

**Keywords:** Mendelian randomization, hip osteoarthritis, lacunar stroke, single-nucleotide polymorphism, genome-wide association studies

## Abstract

Whether hip osteoarthritis (OA) could increase the risk of lacunar stroke (LS) is not well understood. This two-sample Mendelian randomization (MR) study aimed to investigate in depth the effect of genetically predicted hip OA on LS risk. Hip OA-related instrumental variables (IVs) were selected from a genome-wide association study (GWAS) of 393,873 individuals. The summary data of LS were obtained from a GWAS meta-analysis, including 16,030 cases and 248,929 controls. We used the inverse-variance weighted (IVW) as the primary MR analysis method. Moreover, the weighted-median, MR-Egger regression, and the MR pleiotropy residual sum and outlier (MR-PRESSO) test were supplementary methods. The sensitivity analysis was performed using the leave-one-out test. We identified the positive causal relationship between hip OA and the risk of LS (odds ratio [OR] = 1.20, 95% confidence interval [CI]: 1.07, 1.36; *p* = 0.002 using the IVW method). The weighted median method provided similar results. There was no evidence of directed pleiotropy, and sensitivity analysis results were stable, suggesting the robustness of our study. This study showed a causal effect of hip OA on the risk of LS, and more efforts should be made to explore the potential mechanisms in the future.

## 1. Introduction

Lacunar stroke (LS) is a small infarction (less than 2 cm in diameter) that usually occurs from a single perforating artery [1,2]. It contributes to approximately one-quarter of ischemic strokes and about 20% of all strokes [1,2]. It was relatively difficult to identify cases with LS because stroke subtyping is usually conducted according to the clinical ground and non-specific computed tomographic (CT) information [3]. Due to the small size of lacunar infarcts, they are asymptomatic most of the time [4]. Moreover, some researchers pointed out that silent LS could be found in many healthy elderly people [5,6]. It is vital to discriminate LS from other stroke subtypes because of the different etiologies and related mechanisms, indicating that prevention and treatment methods might differ [7,8]. Recently, it has been strengthened that the prognosis after LS is far from benign because of the 20–25% recurrence rate and mortality in the next several years [6]. LS patients would have increased risks of developing cognitive impairment, dementia, etc. [2,9]. Unfortunately, therapy targeting LS is limited and lagged [10]. Thus, early prevention and timely detection would significantly improve the prevention of LS and relatively more severe complications.

Hip osteoarthritis (OA) is a common degenerative joint disease and represents one of the leading causes of pain and disability among the elderly [11,12,13,14]. To date, most clinical research has focused on knee or combined hip and knee OA, with relevant findings generally extrapolated to the hip OA [12,15,16]. There were many differences between hip and knee OA concerning the incidence rate, clinical symptoms, anatomical physiology, treatment measures, and clinical management [16]. Thus, more research into hip OA would be needed to update the relevant existing speculative evidence.

Recently, several observational studies yielded that hip OA was a risk factor for stroke [17,18]. One study with 221,807 individuals from the United Kingdom found that hip OA was associated with an increased risk of stroke (hazard ratio (HR) = 1.21, 95% confidence interval (CI): 1.13–1.31) [18]. In 2020, a large cohort study with more than 320,000 participants confirmed the positive relationship, and the findings showed that hip OA was related to a 1.20-fold increase in the incidence of stroke [17]. Furthermore, in 2022, in order to overcome the shortcomings of the traditional epidemiological methods (e.g., reverse causality), Zhao et al. used the Mendelian randomization (MR) method, which uses genetic variants (single nucleotide polymorphisms, SNPs) to explore a causal relationship [19,20]. They found that a higher risk of hip OA was significantly associated with overall stroke and ischemic stroke [19].

However, existing research evidence for the association between hip OA and LS is still lacking. Therefore, a two-sample MR analysis was performed in this study to investigate whether genetic susceptibility to hip OA is causally related to the risk of LS.

## 2. Materials and Methods

### 2.1. Study Design and Data Sources

**Figure 1:**
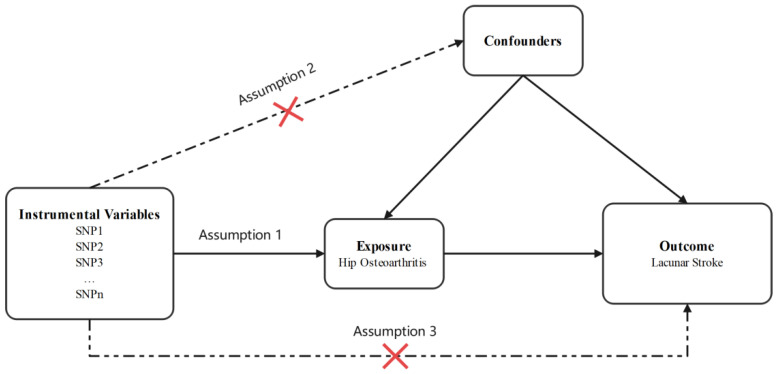
Design flow chart of the present study. Assumption 1: Instrumental variables should be robustly associated with exposure. Assumption 2: Instrumental variables should not be associated with any confounders. Assumption 3: Instrumental variables influence the outcome through the exposure, not through other pathways.

As the data used in this study were derived from published studies and public databases, no additional ethical approval or consent was needed. The flow chart of this study design is shown in Figure 1.

### 2.2. Data Sources

The hip OA summary-level data were obtained from a meta-analysis of genome-wide association studies (GWAS) involving 393,873 samples (15,704 cases and 378,169 controls), which were available at (https://gwas.mrcieu.ac.uk/, accessed on 15 July 2022). The details of this project have been described in previously published studies [21]. The summarized data for genetic variants of LS were obtained from a meta-analysis of GWAS (including previous GWAS from Europe, the USA, and Australia, and additional cases and controls from the UK DNA Lacunar Stroke Studies and the International Stroke Genetics Consortium), including 225,419 samples (6030 LS cases and 248,929 controls), available at (https://gwas.mrcieu.ac.uk/, accessed on 15 July 2022). Details of this project have been described elsewhere [22].

To reduce potential biases from population stratification, all population data in this study were restricted to European ancestry.

### 2.3. Selection of the Genetic Instrumental Variables (IVs)

Genetic variants associated with hip OA were obtained from the UK Biobank and arcOGEN cohort meta-analyses. The IVs were extracted according to the genome-wide association significance threshold (*p* < 5 × 10^−8^ indicated a strong correlation between SNPs and hip OA), and 27 SNPs were selected. All 27 SNPs remained according to the screening criteria (the linkage disequilibrium *r*^2^ > 0.001, and the distance between each other SNPs < 10,000 kb) [23]. Then, in the summary data for LS, one IV was removed because the proxy SNP could not be found. After data harmonization for hip OA and LS, four palindromic IVs were removed because they were palindromic with intermediate allele frequencies.

The *F*-statistic and *R*^2^ for the exposure variance interpreted by each SNP were further calculated to find the weak IVs biases in our MR study [24]. Genetic variants with *F*-statistic < 10 were considered weak IVs and were not included in our MR analysis [25]. The *F*-statistic value of each SNP was more than 10. Details of the selected IVs are presented in Table S1.

### 2.4. Statistical Analyses

All statistical analyses of this MR study were conducted using R version 4.5.0 (http://www.r-project.org (accessed on 15 July 2022)). The related R packages used in our MR study included the TwoSampleMR [26] and MR-PRESSO [27].

The inverse-variance weighted (IVW) method was used as the primary analysis to assess the causal impact of hip OA on the outcome (LS) [28]. Additionally, Cochran’s Q-test was used to assess heterogeneity between SNPs in the IVW method; *p* < 0.05 was considered heterogeneity [29]. Other MR analysis methods, including the weighted median method, the MR-Egger method, and the MR pleiotropy residual sum and outlier (MR-PRESSO) test, were performed as supplementary analyses. The weighted median method could provide robust causal estimates, even though nearly 50% of the relevant information was provided by invalid Ivs [30]. The MR-Egger method could be used to assess the pleiotropy of IVs [31]. The slope of the MR-Egger regression could be used to calculate a causal estimate adjusted for the presence of directed pleiotropy [31,32]. The MR-PRESSO method was applied to detect horizontal pleiotropy and correct it via outlier removal [27]. To determine whether our assessment results were driven by specific SNPs with a significant impact, the leave-one-out sensitivity analysis was performed, removing one SNP in turn and performing the IVW method on the remaining SNPs [33]. We scanned each SNP for its potential secondary phenotypes using the GWAS catalog (http://www.ebi.ac.uk/gwas (accessed on 15 July 2022)) and excluded the SNPs associated with traits other than hip OA (Table S1), and performed the relevant MR analyses again.

The associations between genetically predicted hip OA and LS risk were expressed by the odds ratio (OR) and its 95% confidence intervals (CI). *p* < 0.05 was considered statistically significant. To visualize the MR results, forest plots and scatter plots of SNP-related hip OA and LS risk were drawn using the MR platform-based data analysis function.

## 3. Results

A two-sample MR study was used to assess the causal relationship between hip OA and the risk of LS. We found causal effects of hip OA on LS (Table 1). The detailed results are as follows.

### 3.1. The Final Instruments in the MR Analyses

In this study, according to the genome-wide significant level (*p* < 5 × 10^−8^) and linkage disequilibrium (*R*^2^ < 0.001), the eligible 27 SNPs were extracted (Table S1). Of the 27 SNPs, rs60890741 was excluded because it was not available in the summary data for LS. After data harmonization for hip OA and LS, four SNPs (rs12901372, rs2929451, rs34687269, and rs7222178) were removed from the relevant MR studies because they were palindromic with intermediate allele frequencies. Then, the *F*-statistics of the remaining 22 SNPs were calculated. All the *F*-statistics were above 10, ranging from 805.45 to 2813.57, indicating that the IVs might have less chance of suffering from weak instrument bias and violation of the first assumption [25,34]. Finally, 22 SNPs were collected as genetic instruments for our MR analyses. The characteristics of the genetic variants associated with hip osteoarthritis and their effects on LS in this study are shown in Table 2.

### 3.2. Association between Hip OA and Risk of Lacunar Stroke

**Figure 2:**
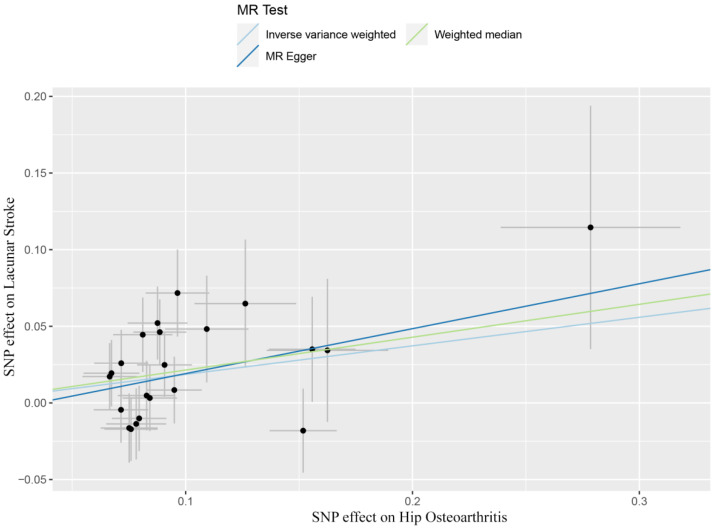
Scatter plots showed the causal effect of hip osteoarthritis on lacunar stroke. SNP, single nucleotide polymorphism; MR, Mendelian randomization.

**Figure 3:**
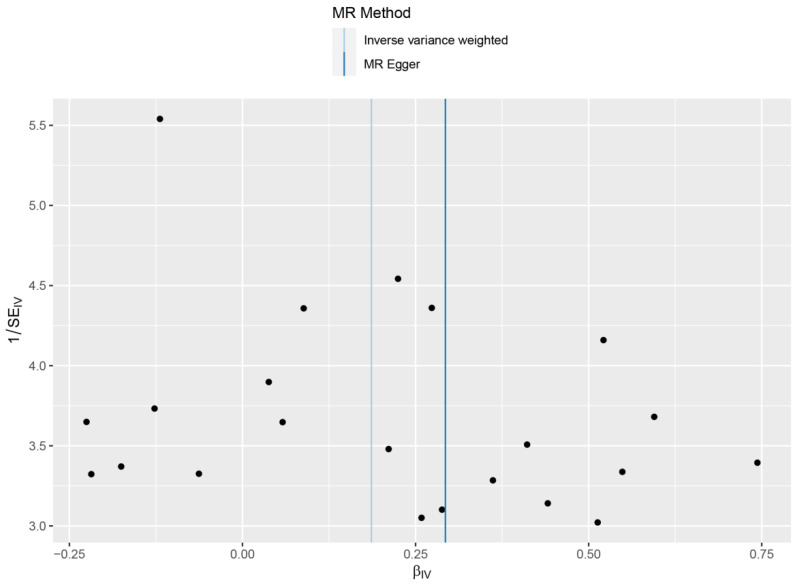
Forrest plots of the causal effects of hip osteoarthritis associated SNPs on lacunar stroke.

By performing IVW analysis, we assessed the relationship between hip OA and LS risk. As shown in Table 1, we confirmed the causal effect of hip OA on the risk of LS (OR = 1.20; 95% CI: 1.07, 1.36; *p* = 0.002), and the scatter plot (Figure 2) also indicated that the risk of LS in hip OA patients increases accordingly. Cochran’s *Q* test showed no heterogeneity (*Q* = 23.35; *p* = 0.325; Figure 3). Moreover, the results of the weighted median method (OR = 1.24; 95% CI: 1.05, 1.46; *p* = 0.012) were substantially similar to those of IVW, further confirming the causal association between hip OA and LS (Table 1). In Table 1, the *p*_for intercept_ of the MR-Egger method was 0.623 (>0.05), which statistically strengthened that the instrumental variables had no horizontal pleiotropy. By the MR-PRESSO method, we did not find outliers for LS.

### 3.3. Sensitivity Analysis

**Figure 4:**
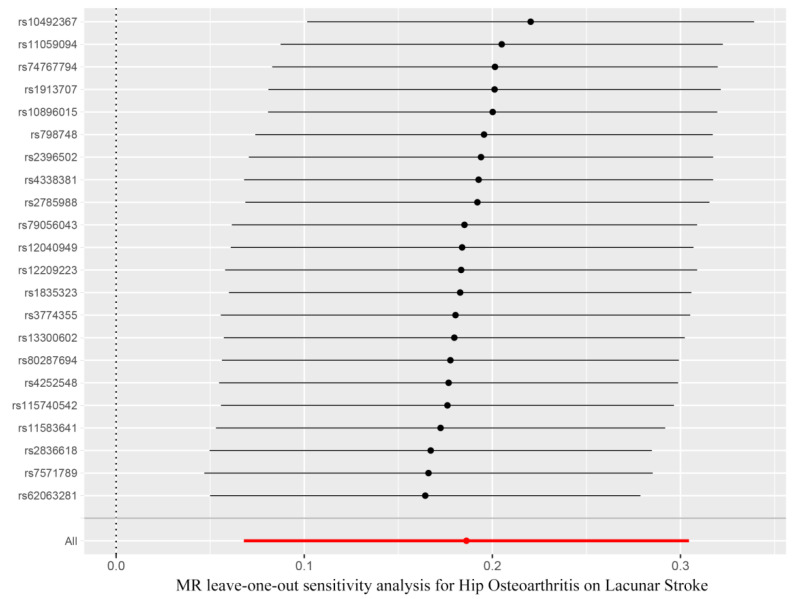
Funnel plot showed no significant heterogeneity among the SNPs. SE, standard error.

In order to analyze the stability of our results, the leave-one-out sensitivity tests were conducted. The analysis results demonstrated that no matter which SNP was removed, the MR analysis results were robust, as shown in Figure 4.

In addition, the GWAS catalog database was searched to exclude SNPs associated with other secondary phenotypes, and we found 7 SNPs associated with other traits (Table S2). After excluding these 7 SNPs, the MR analysis was performed again, and the estimated effect of hip OA on LS risk was similar (OR = 1.18; 95% CI: 1.02, 1.36; *p* = 0.023 by the IVW method; Table S3).

## 4. Discussion

To our knowledge, this is the first study to use a two-sample MR method to explore the causal relationship between genetically predicted hip OA and risk of LS, which has not been demonstrated before.

It has been approved that hip OA could causally affect the risk of ischemic stroke (IS) [19]. However, although accounting for nearly 25% of IS [1,2], LS still differs from other stroke subtypes in etiologies and relevant mechanisms [7,8]. Thus, it is needed to explore the effect of hip OA on LS. In our two-sample MR study, we found the causal relationship between hip OA and LS from a genetic perspective.

The precise mechanisms for the causal relationship between hip OA and LS were unclear. Nevertheless, the potential reasons for the causal association might include inflammation-related factors, changes in physical activities, and diabetes mellitus, central mechanisms of pain secondary to hip OA, etc. Firstly, certain inflammation factors and mechanisms might participate in the occurrence of hip OA, such as the increased circulating levels of interleukin 6 (*IL-6*) in hip OA patients [35]. Wiseman et al. identified that *IL-6* was higher in LS versus non-stroke [7]. Chamorro et al. found a significant relationship between polymorphisms of the *IL-6* gene and LS that could not be discovered in other subtypes of IS [36]. Furthermore, some researchers proposed that *IL-6* and its polymorphisms might be independent risk factors for LS [37]. Thus, inflammation-related factors might support the causal relationship between hip OA and LS. The most common treatment for symptomatic hip OA is total hip replacements (THR) [38]. This surgical procedure might influence the activity of certain inflammation factors and induce inflammation reactions [39]. Secondly, hip OA patients usually reduce strenuous activities, such as walking, because of pains [40]. The increased sedentary behaviors might exacerbate hyperglycemia, the risk of diabetes mellitus, etc. [41]. In addition, it has been reported that the prevalence of diabetes mellitus was higher in LS than in other stroke subtypes, and diabetes mellitus was one of the major risk factors for LS [42,43]. Last but not least, chronic pain distress in hip osteoarthritis patients might increase the risk of LS due to stress response [44,45]. The stress response may increase blood pressure and decrease insulin sensitivity through the hypothalamic-pituitary-adrenal axis and sympathetic nervous system, negatively affecting the vascular system, which might finally increase the risk of LS [19,45,46,47]. In response to emotional stress (including pain distress), the hypothalamic–pituitary–adrenal axis system could also release catecholamines, which may lead to endothelial dysfunction, an important early manifestation of atherosclerosis [46,47]. Carotid atherosclerosis has been approved as an essential risk factor for LS [48]. Although the above explanations are biologically plausible, more studies with elaborate designs are needed to explore and confirm the mechanism of the causal effect of hip OA on LS.

Our present study could overcome some shortcomings of traditional epidemiological studies, including confounding and reverse causation [49]. The second strength is that multiple MR analysis methods were used to obtain more convincing results. Thirdly, the instrumental variables were selected from the recent GWAS studies with larger sample sizes, which could maximize the statistical power. Finally, the leave-one-out sensitivity analysis was performed to analyze the robustness of our results. In addition, to further assess our findings’ robustness, we conducted the secondary MR analyses by excluding the SNPs related to potential secondary phenotypes. Similar results were achieved, which provides additional confidence in the causal relationship between hip OA and LS risk. However, this study still has some limitations. Since our study only included European ancestry participants, the results do not apply to extrapolation to other ethnicities. Therefore, further research with a more diverse population is needed.

## 5. Conclusions

In summary, our study showed a causal effect of hip OA on the risk of LS. The findings might provide new clues for explicating the actual association between hip OA and LS risk.

## Figures and Tables

**Table 1:** Effect estimates of the associations between hip osteoarthritis and risk of lacunar stroke.

Method	SNPs (N)	OR	95%CI	MR *p*-Value	Heterogeneity *Q/p*-Value	Pleiotropy Intercept *p*-Value
IVW	22	1.20	1.07~1.36	0.002	23.35/0.325	
Weighted median	22	1.24	1.05~1.46	0.012		
MR Egger	22	1.34	0.87–2.07	0.203		0.623 ^a^
MR-PRESSO	22	/	/	0.354 ^b^		

SNP, single nucleotide polymorphism; OR, odds ratio; CI, confidence interval; IVW, inverse-variance-weighted; MR, Mendelian randomization; MR-PRESSO, MR pleiotropy residual sum and outlier. ^a^ *p*-value of the intercept from MR Egger regression analysis. ^b^ *p*-value of MR-PRESSO global test.

**Table 2:** Characteristics of the genetic variants associated with hip osteoarthritis and their effects on lacunar stroke (22 SNPs).

SNP	Chr	Position	Effect Allele	SNPs-Hip Osteoarthritis			SNPs-Lacunar Stroke		
				β	SE	*p*-Value	β	SE	*p*-Value
rs10492367	12	28014970	T	0.15	0.01	1.25 × 10^−24^	−0.02	0.03	0.51
rs10896015	11	65323725	A	−0.08	0.01	2.74 × 10^−9^	0.01	0.02	0.56
rs11059094	12	122606837	T	0.08	0.01	7.38 × 10^−11^	−0.02	0.02	0.41
rs115740542	6	26123502	C	0.13	0.02	1.60 × 10^−8^	0.06	0.04	0.12
rs11583641	1	183906245	T	−0.08	0.01	5.57 × 10^−10^	−0.04	0.02	0.07
rs12040949	1	150447462	T	−0.07	0.01	2.83 × 10^−8^	−0.02	0.02	0.43
rs12209223	6	76164589	A	0.16	0.02	3.88 × 10^−16^	0.04	0.03	0.31
rs13300602	9	129412938	G	0.07	0.01	1.65 × 10^−9^	0.03	0.02	0.23
rs1835323	2	43512130	T	−0.07	0.01	4.56 × 10^−8^	−0.02	0.02	0.37
rs1913707	4	13039440	G	−0.08	0.01	2.96 × 10^−11^	0.01	0.02	0.64
rs2396502	6	45357699	C	0.08	0.01	2.12 × 10^−12^	0.00	0.02	0.88
rs2785988	1	219744138	A	0.08	0.01	7.30 × 10^−11^	0.00	0.02	0.83
rs2836618	21	40048295	A	0.09	0.01	3.20 × 10^−11^	0.05	0.02	0.03
rs3774355	3	52817778	A	0.09	0.01	8.20 × 10^−14^	0.02	0.02	0.23
rs4252548	19	55879672	T	0.28	0.04	1.96 × 10^−12^	0.11	0.08	0.15
rs4338381	1	103572927	G	−0.10	0.01	4.37 × 10^−15^	−0.01	0.02	0.70
rs62063281	17	44038785	G	0.10	0.01	5.30 × 10^−12^	0.07	0.03	0.01
rs74767794	1	184006128	G	−0.08	0.01	2.56 × 10^−9^	0.02	0.02	0.47
rs7571789	2	70714793	C	−0.09	0.01	3.26 × 10^−14^	−0.05	0.02	0.03
rs79056043	12	59289598	G	0.16	0.03	1.33 × 10^−9^	0.03	0.05	0.46
rs798748	4	1716770	C	0.07	0.01	2.50 × 10^−9^	0.00	0.02	0.83
rs80287694	6	55636940	G	0.11	0.02	2.66 × 10^−9^	0.05	0.03	0.17

SNP, single nucleotide polymorphism; SE, standard error.

## Data Availability

All data are available on reasonable request from corresponding author.

## Supplementary Materials

The following supporting information can be downloaded at: https://www.mdpi.com/article/10.3390/genes13091584/s1, Table S1: Selecting instrumental variables related to hip osteoarthritis by GWAS threshold (*p* < 5 × 10^−8^) (27 SNPs); Table S2: The secondary phenotypes of the instrumental variables used for hip osteoarthritis (from the GWAS catalog); Table S3: Effect estimates of the associations between hip osteoarthritis and risk of lacunar stroke after excluding potential secondary phenotypes SNPs.
