# Supplementary material for: Hip Osteoarthritis and the Risk of Lacunar Stroke: A Two-Sample Mendelian Randomization Study
Source: Genes (Basel). 2022 Sep 3;13(9):1584. doi: 10.3390/genes13091584 (PMC9498627; doi:10.3390/genes13091584)
Supplement: Supplementary file 1 [file genes-13-01584-s001.zip › genes-1868745-supplementary.pdf]

Supplementary Materials

Table S1 Selecting instrumental variables related to hip osteoarthritis by GWAS threshold (*p*-value < 5 × 10⁻⁸) (27 SNPs)

**SNP**	**Position**	**Chr**	**EA**	**OA**	**EAF**	**β**	**SE**	***p*-value**	***R*²**	***F***
rs12040949	150447462	1	T	C	0.38	-0.07	0.01	2.83×10^{-8}	0.002	825.99
rs11583641	183906245	1	T	C	0.28	-0.08	0.01	5.57×10^{-10}	0.003	1038.98
rs4338381	103572927	1	G	A	0.37	-0.10	0.01	4.37×10^{-15}	0.004	1660.63
rs74767794	184006128	1	G	A	0.32	-0.08	0.01	2.56×10^{-9}	0.002	964.45
rs2785988	219744138	1	A	C	0.30	0.08	0.01	7.30×10^{-11}	0.003	1134.79
rs7571789	70714793	2	C	T	0.52	-0.09	0.01	3.26×10^{-14}	0.004	1548.47
rs1835323	43512130	2	T	C	0.34	-0.07	0.01	4.56×10^{-8}	0.002	805.45
rs3774355	52817778	3	A	G	0.36	0.09	0.01	8.20×10^{-14}	0.004	1498.94
rs798748	1716770	4	C	T	0.62	0.07	0.01	2.50×10^{-9}	0.002	952.72
rs1913707	13039440	4	G	A	0.39	-0.08	0.01	2.96×10^{-11}	0.003	1185.45
rs12209223	76164589	6	A	C	0.10	0.16	0.02	3.88×10^{-16}	0.004	1777.66
rs2396502	45357699	6	C	A	0.60	0.08	0.01	2.12×10^{-12}	0.003	1342.89
rs115740542	26123502	6	C	T	0.07	0.13	0.02	1.60×10^{-8}	0.002	864.02
rs80287694	55636940	6	G	A	0.11	0.11	0.02	2.66×10^{-9}	0.002	946.25
rs60890741	130768503	8	CA	C	0.14	-0.11	0.02	4.50×10^{-9}	0.003	1098.10
rs2929451	9085295	8	T	A	0.51	-0.07	0.01	3.11×10^{-9}	0.002	942.12
rs13300602	129412938	9	G	A	0.45	0.07	0.01	1.65×10^{-9}	0.003	1002.37
rs34687269	119484132	9	T	A	0.47	-0.08	0.01	1.67×10^{-12}	0.003	1342.84
rs10896015	65323725	11	A	G	0.27	-0.08	0.01	2.74×10^{-9}	0.002	950.66
rs10492367	28014970	12	T	G	0.19	0.15	0.01	1.25×10^{-24}	0.007	2813.57
rs79056043	59289598	12	G	A	0.05	0.16	0.03	1.33×10^{-9}	0.003	996.19
rs11059094	122606837	12	T	C	0.48	0.08	0.01	7.38×10^{-11}	0.003	1135.52
rs12901372	67370506	15	G	C	0.47	-0.08	0.01	3.46×10^{-11}	0.003	1205.78

rs62063281	44038785	17	G	A	0.22	0.10	0.01	5.30×10^{-12}	0.003	1272.11
rs7222178	59652282	17	A	T	0.20	0.10	0.01	3.77×10^{-11}	0.003	1173.22
rs4252548	55879672	19	T	C	0.02	0.28	0.04	1.96×10^{-12}	0.003	1319.01
rs2836618	40048295	21	A	G	0.26	0.09	0.01	3.20×10^{-11}	0.003	1170.28

SNP, single nucleotide polymorphism; Chr, chromosome; EA, effect allele; OA, other allele, SE, standard error.

[a] R^2 were calculated using the following formula: 2×MAF×(1-MAF)×Beta^2, where MAF is the minor allele frequency , Beta is the estimated effect on hip osteoarthritis.

[b] F were calculated using the following formula: R^2(N-2)/(1-R^2), where R^2 is the proportion of variance in hip osteoarthritis explained by each instrument and N is the sample size of the GWAS for the hip osteoarthritis association.

Table S2 The secondary phenotypes of the instrumental variables used for hip osteoarthritis (from the GWAS catalog)[a].

SNP	Chr	Position	Trait[a]	*p*-value
rs10896015	11	65556254	Serum alkaline phosphatase levels	1.00×10^{-10}
rs115740542	6	26123274	Serum levels of protein TF	3.00×10^{-26}
			Free cholesterol levels in large LDL	2.00×10^{-16}
			Concentration of large LDL particles	6.00×10^{-11}
			Phospholipid levels in large LDL	1.00×10^{-13}
			Estimated glomerular filtration rate (creatinine)	3.00×10^{-10}
			Cholesterol levels in small LDL	2.00×10^{-14}
			Phospholipid levels in LDL	2.00×10^{-14}
			Ratio of linoleic acid to total fatty acids	$6.00E \times 10^{-9}$
			Phospholipids to total lipids ratio in medium HDL	2.00×10^{-12}
			Free cholesterol levels in LDL	3.00×10^{-17}
			Free cholesterol levels in medium LDL	3.00×10^{-17}
			Phospholipid levels in small LDL	3.00×10^{-16}
			Free cholesterol levels in small LDL	4.00×10^{-19}
			Total lipid levels in small LDL	3.00×10^{-14}
			Serum levels of protein NAALAD2	4.00×10^{-24}
			Total esterified cholesterol levels	5.00×10^{-12}
			Total cholesterol levels	1.00×10^{-11}
			Clinical LDL cholesterol levels	2.00×10^{-14}
			Total free cholesterol levels	4.00×10^{-10}
			Sphingomyelin levels	1.00×10^{-12}
			Hemoglobin concentration	2.00×10^{-580}
			Waist circumference adjusted for body mass index	$4.00E \times 10^{-8}$
			Skin and soft tissue infections	4.00×10^{-7}
			Apolipoprotein B levels	5.00×10^{-10}
rs12209223	6	75454873	Height	5.00×10^{-25}
			Hip circumference adjusted for BMI	3.00×10^{-18}
rs4252548	19	55368304	Appendicular lean mass	5.00×10^{-35}
			Height	3.00×10^{-68}
			Lung function (FVC)	5.00×10^{-9}
rs4338381	1	103107371	Height	2.00×10^{-6}
rs62063281	17	45961419	Number of sexual partners	4.00×10^{-15}
rs798748	4	1715043	Mean reticulocyte volume	4.00×10^{-15}

Chr, chromosome; SNP, single nucleotide polymorphism.

[a]Traits associated with the SNP according to previous genome-wide association studies.

Table S3 Effect estimates of the associations between hip osteoarthritis and risk of lacunar stroke after excluding potential secondary phenotypes SNPs.

Method	SNPs (N)	OR	95%CI	MR *p*-value	Heterogeneity *Q* / *p*-value	Pleiotropy Intercept *p*-value
IVW	15	1.18	1.02~1.36	0.023	15.70/0.332	
Weighted median	15	1.22	1.01~1.49	0.04		
MR Egger	15	0.99	0.53-1.85	0.969		0.575[a]
MR-PRESSO	15	/	/	0.101[b]		

SNP, single nucleotide polymorphism; OR, odds ratio; CI, confidence interval; IVW, Inverse-variance-weighted; MR, Mendelian randomization; MR-PRESSO, MR Pleiotropy RESidual Sum and Outlier.

[a] The *p*-value of the intercept from MR Egger regression analysis.

[b] The *p*-value of MR-PRESSO global test.
